# Elucidating Semiconducting Properties and Photocatalytic Performance of Surface-Decorated BiVO_4_ for the Removal of Contaminants of Emerging Concern

**DOI:** 10.3390/molecules30112454

**Published:** 2025-06-03

**Authors:** Marin Popović, Suresh Kumar Pandey, Josipa Papac Zjačić, Vladimir Dananić, Marijana Kraljić Roković, Marin Kovačić, Hrvoje Kušić, Andraž Šuligoj, Urška Lavrenčič Štangar, Ana Lončarić Božić

**Affiliations:** 1 Department for Safety and Protection Engineering, Karlovac University of Applied Sciences, Trg Josipa Juraja Strossmayera 9, HR-47000 Karlovac, Croatia; m210p90@gmail.com; 2 Faculty of Chemical Engineering and Technology, University of Zagreb, Trg Marka Marulića 19, HR-10000 Zagreb, Croatia; skpandey@fkit.unizg.hr (S.K.P.); jpapac@fkit.unizg.hr (J.P.Z.); vdanan@fkit.unizg.hr (V.D.); mkralj@fkit.unizg.hr (M.K.R.); mkovacic@fkit.unizg.hr (M.K.); abozic@fkit.unizg.hr (A.L.B.); 3 Department for Packaging, Recycling and Environmental Protection, University North, Trg Dr. Žarka Dolinara 1, HR-48000 Koprivnica, Croatia; 4 Faculty of Chemistry and Chemical Technology, University of Ljubljana, Večna Pot 113, SI-1000 Ljubljana, Slovenia; andraz.suligoj@fkkt.uni-lj.si (A.Š.); urska.lavrencic.stangar@fkkt.uni-lj.si (U.L.Š.); 5 Department of Inorganic Chemistry and Technology, National Institute of Chemistry, Hajdrihova 19, SI-1001 Ljubljana, Slovenia

**Keywords:** BiVO_4_, surface decoration, semiconducting properties, solar photocatalysis, contaminants of emerging concern

## Abstract

Photocatalysis has been shown to be a promising and ecofriendly approach to the removal of contaminants of emerging concern (CECs). However, preventing the recombination of photogenerated charge carriers and achievement of suitable band edge positions are still major challenges to ensuring better performance. Herein, we report the preparation of surface-decorated BiVO_4_ with both a noble metal (Ag) and transition metal (Fe). The structural, morphological, and semiconducting features of the material were examined employing various techniques (XRD, SEM, UV-DRS, PL, and photoelectrochemical tests). The band gap of surface-modified BiVO_4_ is slightly narrower compared to pristine material, which is further validated by HOMO-LUMO gaps obtained through theoretical modeling approaches. The recombination of photogenerated charges was successfully reduced in the case of Ag–Fe–BiVO_4_, as proven by lower PL intensity and increased current density. The comparative photocatalytic degradation of the CECs ciprofloxacin (CIP) and perfluorooctanoic acid (PFOA) was conducted employing pristine BiVO_4_ and its two surface-modified analogues (Ag–BiVO_4_, and Ag–Fe–BiVO_4_) under solar light. Ag–Fe–BiVO_4_ was shown to be the most efficient; however, its effectiveness differed depending on CEC type. Under the same process conditions, degradation of CIP reached 93.9%, while PFOA was degraded only partially (22.9%).

## 1. Introduction

Semiconductor photocatalysis is one of the most promising remediation techniques and has received comprehensive attention over the past several decades, specifically for the removal of persistent organic pollutants (POPs) from the environment and for hydrogen production through water splitting by utilizing solar energy [1,2,3]. To date, several common metal oxide photocatalysts, such as titanium dioxide (TiO_2_), zinc oxide (ZnO), tungsten oxide (WO_3_), cerium oxide (CeO_2_), and tin oxide (SnO_2_), have been employed for the treatment of organic pollutants [4]. Among these, TiO_2_- and ZnO-based photocatalysts have been extensively studied due to their low cost, non-toxic nature, high electron mobility, and strong oxidative potentials. However, the practical applications of both TiO_2_ and ZnO photocatalysts are greatly impeded by their wide band gaps (≈3.2 eV), which enables them to utilize only the UV-A portion of solar light (3–5%); additionally, ZnO is more susceptible to photo-corrosion in aqueous environments, which can negatively affect its long-term stability [5,6]. To harness solar energy effectively and reduce reliance on artificial light sources for photocatalysis, various strategies have been adopted for TiO_2_ structure–properties engineering, including doping/incorporation of noble metals/non-metals or transition metal elements and fabrication of composites spanning over a broad variety of chemical compositions with other semiconducting materials, conductive polymers, and/or carbon-based materials [7,8,9]. Furthermore, the development of efficient visible light-responsive photocatalysts presents a promising alternative. In order to achieve this, a range of photocatalysts has been explored, including inorganic, molecular, and hybrid inorganic/organic materials, to find those that meet the essential criteria [10]. Among the various photocatalysts developed, bismuth-based photocatalysts have garnered significant attention due to their compatibility with a wide range of earth-abundant materials [11]. The valence band (VB) of these semiconductors is primarily composed of Bi 6s and O 2p hybrid orbitals, which results in lower band gaps compared to many other metal oxides that predominantly feature O 2p orbitals. Consequently, most bismuth-based semiconductors exhibit band gaps of less than 3.0 eV, enabling effective absorption of visible light [12].

Several bismuth-containing compounds, including BiOI, BiOBr, Bi_2_WO_6_, Bi_4_Ti_3_O_12_, Bi_2_O_2_CO_3_, Bi_2_MoO_6_, and BiVO_4_ have demonstrated excellent photocatalytic properties for both water purification and energy conversion via water splitting [13,14,15]. Among these, BiVO_4_ stands out due to its numerous advantages, such as being environmentally friendly, resistant to photo-corrosion, non-toxic, and cost-effective. It is an n-type semiconductor with a direct band gap of 2.4 eV [16]. Notably, the valence band edge position of BiVO_4_ is around 2.8 eV vs. RHE, which allows for the oxidation of H_2_O (i.e., OH^−^) to hydroxyl radicals (^●^OH), thereby enhancing the degradation efficiency of organic pollutants. BiVO_4_ has three crystal polymorphs: two tetragonal forms (zirconia and scheelite analogues) and a monoclinic modification with a distorted scheelite structure. The latter form shows higher photocatalytic activity compared to tetragonal polymorphs [17].

In a previous study, Borowska et al. investigated the photocatalytic activity of TiO_2_ for the degradation of sulfamethoxazole (SMX) under natural sunlight and reported only 50% degradation after 4 h [18]. In another study, Ahmed et al. evaluated TiO_2_ (Degussa P25) under a white lamp and UV light. The catalyst achieved 23% SMX degradation under the white lamp, but a significantly higher degradation of 85% under UV light after 7 h [19]. Similarly, Pourmoslemi et al. reported approximately 60% SMX removal using a ZnO catalyst under UV irradiation for 5 h [20]. In contrast, several bismuth-based photocatalysts such as BiOCl and BiVO_4_ have shown much higher photocatalytic efficiency under visible light. For instance, BiOCl achieved 87% degradation of SMX in just 90 min under a solar simulator, while BiVO_4_ degraded 54% of SMX within 60 min under xenon lamp irradiation [21,22]. Overall, these studies suggest that while TiO_2_ and ZnO are more effective under UV light, bismuth-based photocatalysts exhibit superior performance under visible light, making them promising candidates for solar-driven photocatalysis.

However, the photocatalytic performance of BiVO_4_ is still constrained by its limited surface area and high recombination rates of photogenerated excitons: electrons and holes (*e*^−^/*h*^+^) [23]. A variety of strategies, as mentioned above in the case of TiO_2_ modifications, can be applied for BiVO_4_ improvement as well. Hence, noble metal incorporation, ion doping, and the engineering of semiconductor heterojunctions have been explored to overcome these challenges and significantly enhance the photocatalytic activity of BiVO_4_ [24]. Incorporation of noble metals like Ag, Au, and Pt onto the BiVO_4_ surface enhances its absorption capability through Surface Plasmone Resonance and formation of a Schottky barrier. Moreover, these noble metals act as electron sinks, which take excited electrons from the semiconductor and further release them as hot electrons which combine with oxygen and may form anionic superoxide radicals (O_2_^●−^) [15,16,17,18,19,20,21,22,23,24,25,26,27,28]. Coupling with transition metals (e.g., Fe) may lead to the formation of heterojunctions (when present at the surface) or in-gap states between valence bands and conduction bands (when doped into the BiVO_4_ lattice), thus enhancing the tendency of *e*^−^/*h*^+^ separation and consequently improving visible light absorption capacity and, eventually, photocatalytic activity [29,30].

In the present study, we aim to improve the semiconducting properties of BiVO_4_ by modifying its surface through photodeposition of Ag alone and both Ag and Fe, which were compared to pristine BiVO_4_ in the form of isotype homojunctions [31,32]. The material characterization was assessed by a combined computational (DFT) and experimental approach targeting structural and optoelectronic properties. The photocatalytic performance of synthetized materials was evaluated and compared in the solar-driven treatment of two contaminants of emerging concern (CECs); perfluorooctanoic acid (PFOA) and ciprofloxacin (CIP). These two CECs were selected due to their high persistence in water environments [33,34], as well as their susceptibility to reductive degradation [35,36]. For example, PFOA can undergo almost exclusively reductive degradation, i.e., cleavage of C–F bonds, while negligible oxidation may occur in terms of braking C–C bonds and eventually shortening alkyl chains and creating smaller perfluorinated by-products, characterized by their persistence in the environment [37]. CIP may undergo both oxidative and reductive degradation mechanisms [35,38,39]; however, it was found that the reductive one is more favorable to TiO_2_ photocatalysis [35]. Hence, these CECs are excellent representative candidates for exploring the reductive potential of surface-decorated BiVO_4_.

## 2. Results and Discussion

Firstly, we describe the modeling of our materials and studying the materials’ properties, i.e., the HOMO-LUMO gap. Our theoretical modeling approach included clusters composed of BiVO_4_ units, where BiVO_4_ was present as an amorphous material due to the fact that the addition of silver and iron disrupted its crystallinity. In the structures thus obtained, we observed the HOMO-LUMO gap as the main measure of cluster reactivity. The results are shown in Table 1. As might be expected, the HOMO-LUMO gap does not show any strict dependence on the cluster size. This gap is a statistical quantity that varies in a random manner within a certain range. The average gap value for a cluster of “pure” BiVO_4_ is 3.33 eV with a standard deviation of 0.5 eV. When we add two silver atoms to each of the optimized clusters and re-optimize such clusters, we get an average value of 1.72 eV with a standard deviation of 0.5 eV. This gives us the right to claim that the addition of silver lowers the gap while maintaining almost the same standard deviation. The same applies to the addition of iron, with the difference that iron also lowers the HOMO-LUMO gap, but not to the same extent as silver. The average value of the gap with the addition of iron is 2.4 eV with a standard deviation of 0.6 eV. 

**Figure 1:**
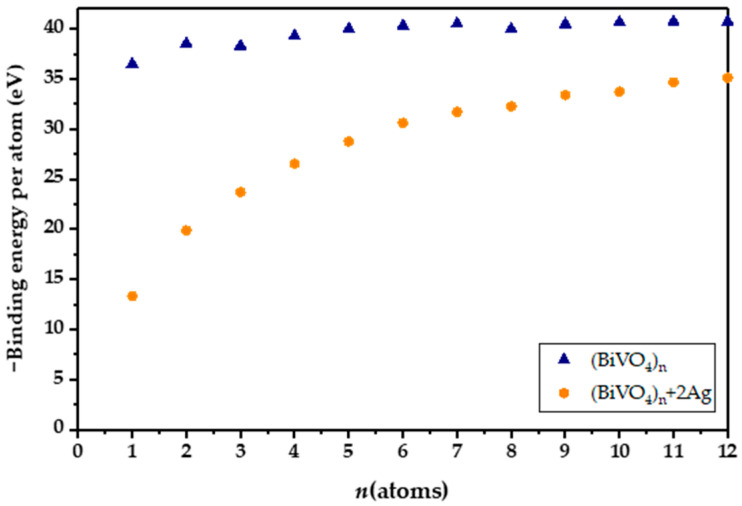
Binding energies for BiVO_4_ and doped amorphous clusters determined by Equation (1).

**Figure 2:**
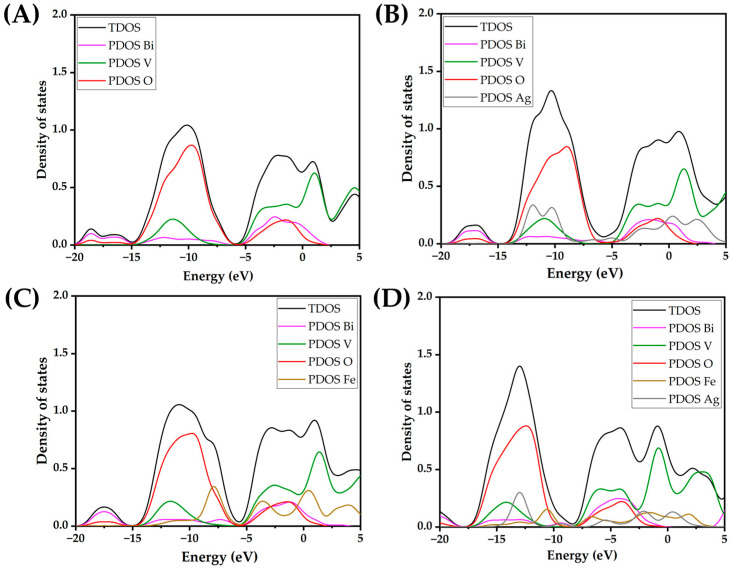
Total density of states (TDOS) and partial density of states (PDOS) for atomic species for (**A**) (BiVO_4_)_4_, (**B**) (BiVO_4_)_4_ + 2Ag, (**C**) (BiVO_4_)_4_ + 2Fe, and (**D**) (BiVO_4_)_4_ + Ag + Fe.

Introducing both Ag and Fe yields a gap of 2.11 eV, which is higher than for a solely Ag-doped nanocluster, and lower than for an Fe-doped nanocluster. Experimental studies have confirmed that doping BiVO_4_ with Ag results in narrowing of the BiVO_4_ band gap, caused by simultaneous narrowing of the valence and conduction bands [40]. However, in our DFT study, the atomistic ratio of Ag and Fe is very high, leading to marked effects on the band gaps, which are not observable in practice. On the other hand, Fe-3d states are below the conduction band of BiVO_4_ and therefore do not result in as dramatic an effect as Ag [41]. All in all, we can say that quantum mechanical calculations clearly show the pronounced effect of lowering the HOMO-LUMO gap in the BiVO_4_ amorphous sample with the addition of Ag and Fe atoms. The binding energy per atom calculations for the (BiVO_4_)_n_ clusters have revealed that the relative increase in stability, i.e., the magnitude of binding energy, does not increase substantially after the tetramer. Moreover, the binding energies per atom for the clusters ranging from *n* = 7 to 12 reveal an asymptotic trend, implying bulk-like properties. However, with the inclusion of heteroatoms, i.e., Ag or Fe, or combination thereof, the per-atom binding energy drops substantially. In addition, the per-atom binding energy for clusters that are smaller than *n* = 7 is substantially smaller in magnitude (Figure 1). This indicates potentially lower stability of the doped clusters, and potentially higher reactivity. In order to elucidate their reactivity, total densities of states (TDOS) and partial densities of states (PDOSs) were plotted for the tetramer (*n* = 4) nanoclusters, as shown in Figure 2. We can readily observe that the HOMO levels for the Fe- and Ag-doped clusters are higher in energy, i.e., closer to the vacuum level. It is important to note that the Ag-doped tetramer has the most positive HOMO. The shifting of the HOMO level implies greater oxidation potential of the photogenerated holes, which in turn could directly enhance photocatalytic activity through direct oxidation and the formation of ^●^OH radicals. On the other hand, Ag and Fe have lowered the LUMO as well, which is most evident for Ag, whereby Ag doping results in a LUMO electron state within the unmodified BiVO_4_ HOMO-LUMO gap. This in turn increases the reduction potential of the photogenerated electrons, in addition to narrowing the band gap. Such a shift is highly favorable from the standpoint of PFAS photodegradation, since superoxide radical formation is promoted. Furthermore, since the additional state is near the LUMO level of the unmodified BiVO_4_ nanocluster, we could assume that this state acts as a shallow electron trap. In particular, shallow trapping states enhance photocatalytic activity by suppressing photogenerated charge recombination [42,43].

As for the Ag- and Fe-co-doped nanocluster, its electronic properties are a result of the complex interplay between the dopants and BiVO_4_. The Ag- and Fe-doped tetramer cluster has displayed a markedly more negative HOMO level in relation to the Ag- or Fe-doped nanoclusters alone. However, the Ag- and Fe-doped nanocluster displays a more negative LUMO level and also a narrowed HOMO-LUMO gap. Thus, we could expect the Ag- and Fe-doped nanocluster to display a lower optical bandgap and also potentially improved photoactivity for the removal of PFAS and not only pharmaceuticals, as already proven in a previous study [30,31].

**Figure 3:**
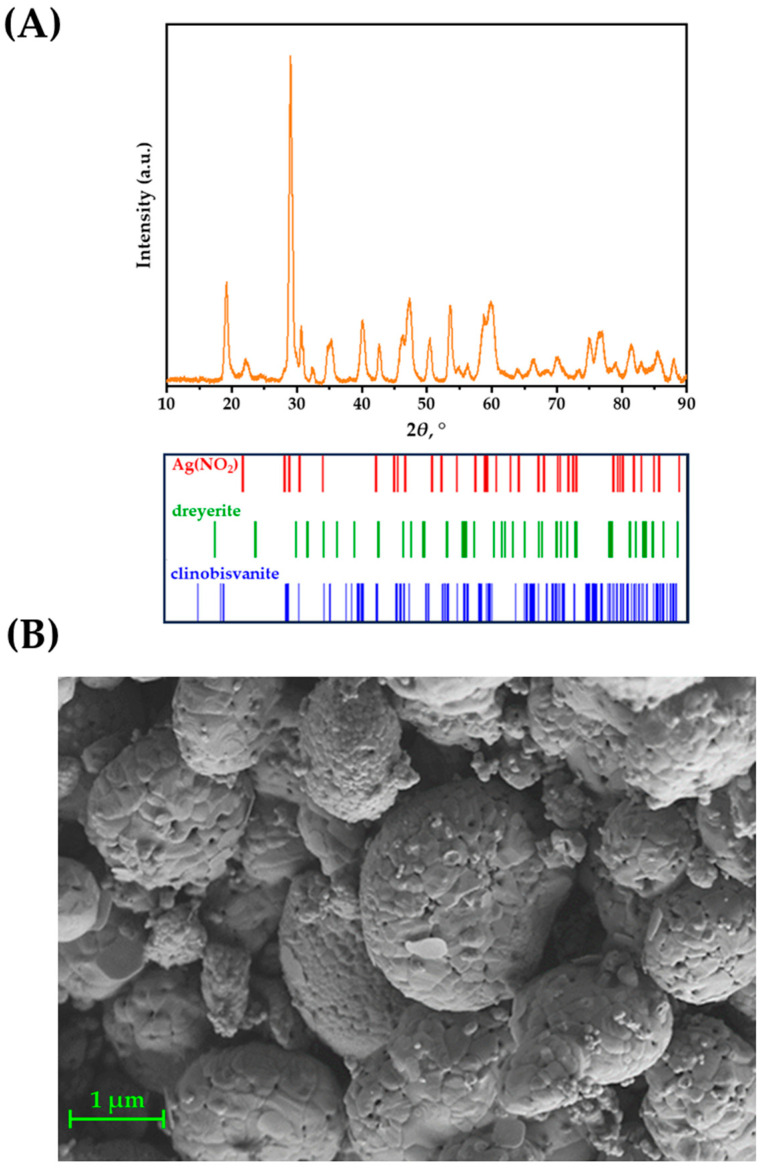
(**A**) Powder diffraction (PXRD) for the Ag–Fe–BiVO_4_ sample, along with corresponding peak positions for the identified phases and (**B**) SEM analysis of Ag–Fe–BiVO_4_.

The Ag–Fe–BiVO_4_ material was characterized for its structural and morphological properties by employing XRD and SEM techniques. The obtained results were compared with those for single-doped materials studied previously [30]. As can be seen from Figure 3A, presenting the results of powder XRD analysis, the crystalline structure typical of a synthesized BiVO_4_ iso-type homojunction [31] is retained upon doping, or more correctly surface deposition, of material by Ag and Fe. Moreover, Ag(NO_2_) has been identified, suggesting the ability of the iso-type homojunction BiVO_4_ to reduce Ag(NO)_3_ under illumination, which presumably occurred on {010} facets [44]. The presence of metallic Ag was not identified by XRD; however, the diffraction signal corresponding to the Ag nanoparticles may have been hidden within the background signal and/or overlapped with the peaks of other phases. No crystalline iron phases were identified; however, the sample did exhibit a somewhat higher background intensity when the diffractogram was recorded without using the XRF suppression mode of the 1D detector. This higher background intensity is possibly a side effect of X-ray fluorescence in an amorphous Fe phase, especially taking into account the strong K-shell absorption edge of Fe and its high fluorescence yield.

SEM/EDX analysis confirmed results obtained previously for the single-doped BiVO_4_ [30]; the morphology of material is preserved. Hence, globular aggregates of roughly 1 µm in diameter formed by the fusion of smaller individual particles can be observed in Figure 3B. It should be noted that Ag and Fe species are not observable on the BiVO_4_ surface in SEM micrograph, which is to some extent expected due to small Ag(NO_2_) crystallites and since no Fe-containing phase was identified according to XRD analysis. However, the results of our previous study on Fe–BiVO_4_ clearly showed that, although Fe was not detected by XRD, SEM/EDX, and XPS techniques, it was undoubtedly present since the activity was significantly improved compared to pristine BiVO_4_ [30]. Hence, the same can be expected in the case of double-doped BiVO_4_ prepared here.

**Figure 4:**
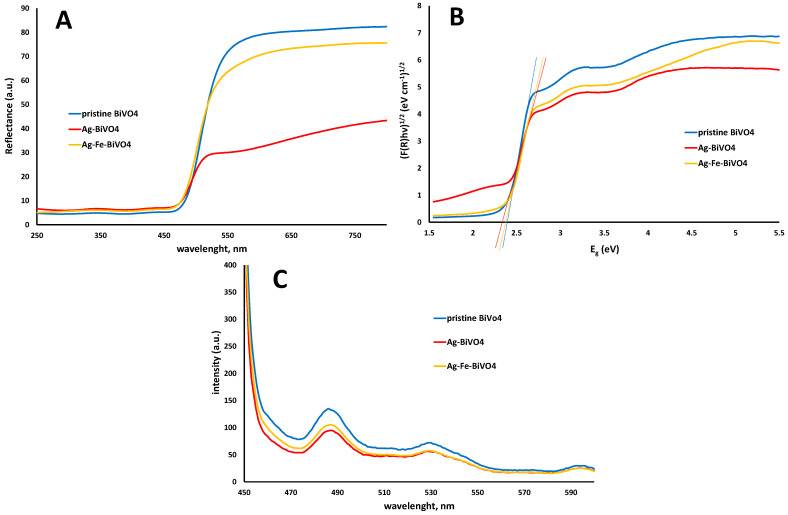
Diffuse reflectance spectra of semiconducting materials: pristine BiVO_4_, Ag–BiVO_4_, and Ag–Fe–BiVO_4_ (**A**), corresponding plots of transformed Kubelka–Munk function vs. the energy of the light (**B**), and PL spectra (**C**).

The semiconducting properties of pristine BiVO_4_ and doped (Ag–BiVO_4_ and Ag–Fe–BiVO_4_) materials were studied as well. In Figure 4 are presented their diffuse reflectance spectra (A), corresponding plots of transformed Kubelka–Munk function vs. the energy of the light (B), and PL spectra (C).

As can be seen from Figure 4A,B doping of BiVO_4_ with Ag or with both Ag and Fe did not significantly influence absorption spectra in terms of reflectance and corresponding band gap values. Hence, Ag doping decreased the band gap by 0.02 eV from 2.40 eV in the case of pristine BiVO_4_ to 2.38 eV in the case of Ag–BiVO_4_. Co-doping with Ag and Fe resulted in a band gap of 2.30 eV (Figure 4B). The reason can be found in the fact that both heteroatoms, Ag and Fe, are surface deposited and in such cases Ag serves as an electron sink and does not directly influence light absorption properties, i.e., band gaps. On the other hand, Fe may form at the surface heterojunction and slightly influence light absorption properties, thus resulting in slightly decreased band gaps. However, the positive influence of Ag and Fe surface deposition can be seen from the PL results (Figure 4C). All three materials showed a specific emission peak at 485 nm, although with different intensities. In both cases of doped materials, Ag–BiVO_4_ and Ag–Fe–BiVO_4_ exhibited lower emission intensities compared to the one recorded for pristine BiVO_4_. Hence, it can be expected that all three materials would have similar abilities to absorb visible light, while doped analogues may show higher photocatalytic activity due to the fact that a lower intensity PL emission peak means a higher *e*^−^/*h*^+^ separation probability [45].

**Figure 5:**
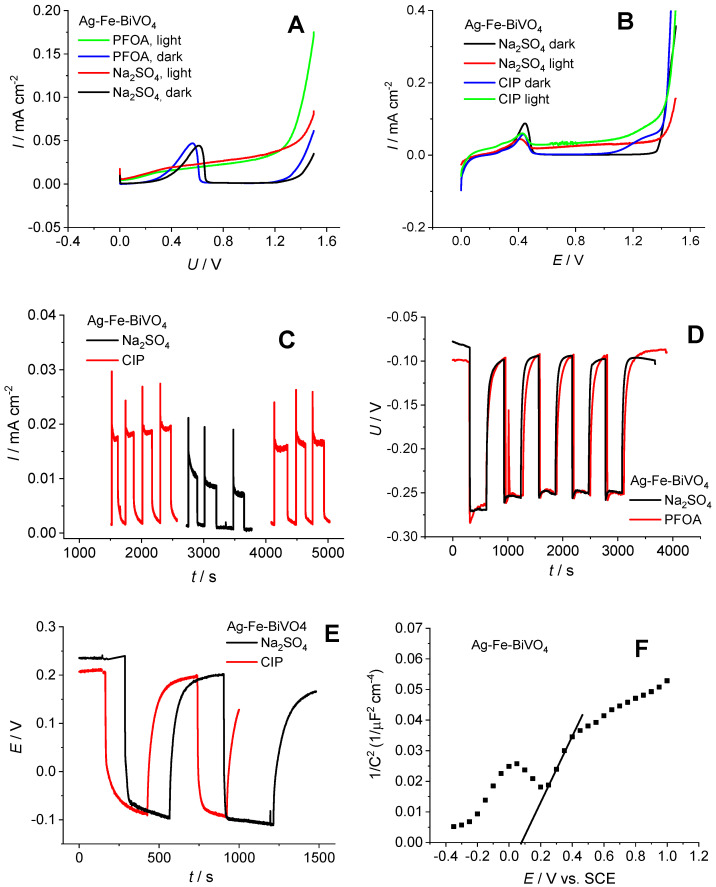
Linear sweep voltammetry (LSV) responses in the dark and under illumination for Fe–Ag–BiVO_4_ electrode examined in Na_2_SO_4_ and PFOA (**A**) and Na_2_SO_4_ and CIP (**B**); chronoamperometry response in Na_2_SO_4_ and CIP (**C**); OCP monitoring for both PFOA and CIP (**D**,**E**); and Mott–Schottky analysis (**F**).

To gain better insight into the behavior of Fe–Ag–BiVO_4_ and its semiconducting properties in systems with targeted CECs, the electrochemical characterization of Fe–Ag–BiVO_4_ was conducted separately in 0.5 M Na_2_SO_4_ containing CIP and PFOA. The measurements in the presence of PFOA were conducted in a two-electrode system in order to prevent strong adsorption of PFOA at the reference electrode [46], while measurements in the presence of CIP were conducted in a three-electrode system. Linear sweep voltammetry (LSV) responses in the dark and under illumination were recorded (Figure 5A,B). The characteristic current peak registered between 0 V and 0.5 V is related to the oxidation of Ag nanoparticles on the surface of BiVO_4_ [30]. The higher currents of the illuminated electrode compared to dark responses indicate good photoactivity of Ag–Fe–BiVO_4_. The current values in CIP are about 0.015–0.04 mA cm^−2^ and in PFOA are about 0.03–0.05 mA cm^−2^, which correspond to the values of Ag–BiVO_4_ and Fe–BiVO_4_ [30]. In a previous investigation, it was shown that LSV response can be influenced by the presence of organic pollutants; moreover, degradation rate can be predicted by this information [47]. The results obtained in this study, in the case of PFOA, indicate that there is no significant difference between the responses obtained in the presence and absence of PFOA, suggesting the stability of PFOA in the oxidation process. On the other hand, the current increased in the presence of CIP, thus indicating hole scavenger properties which contribute to a lower recombination rate and consequent current increase. In addition, current peaks above 1 V for dark and illuminated Ag–Fe–BiVO_4_ electrodes indicate electrochemical oxidation of CIP, which was not noticed for PFOA. This provides additional evidence of the high stability of PFOA. In order to additionally test the hole scavenger properties of CIP, chronoamperometry (CA) measurement was conducted at 0.6 V (Figure 5C). From the consecutive examination of Ag–Fe–BiVO_4_ electrodes in Na_2_SO_4_ and CIP it is obvious that CIP increases photocurrent and, moreover, stabilizes the current response over time. By monitoring OCP (Figure 5D,E), it is evident that upon illumination the potential changes to more negative values, indicating its photoactivity and being characteristic of n-type semiconductors. When illumination is terminated, potential shifts towards the initial value. This change is fast that was not observed for unmodified BiVO_4_ [30,48]. In previous investigations, such behavior was noticed for Ag-modified BiVO_4_ [24], but not for Fe-modified BiVO_4_. Therefore, it can be concluded that Ag dominates the overall OCP response of Ag–Fe–BiVO_4_. OCP monitoring in the presence and absence of CIP or PFOA indicated no significant difference in response. The Mott–Scottky response reveals the flat band potential (E_FB_) of Ag–Fe–BiVO_4_, which indicates the position of conduction bands. E_FB_ was determined from the *X*-axis section of the Mott–Schottky plots according to the equation *E*_FB_ = (*E* − *k*BT/e)y = 0 and the value of E_FB_ amounts to 0.082 V vs. SCE. It is obvious that the tested material does not show strong reduction power and for this reason rather low degradation of PFOA can be expected.

**Figure 6:**
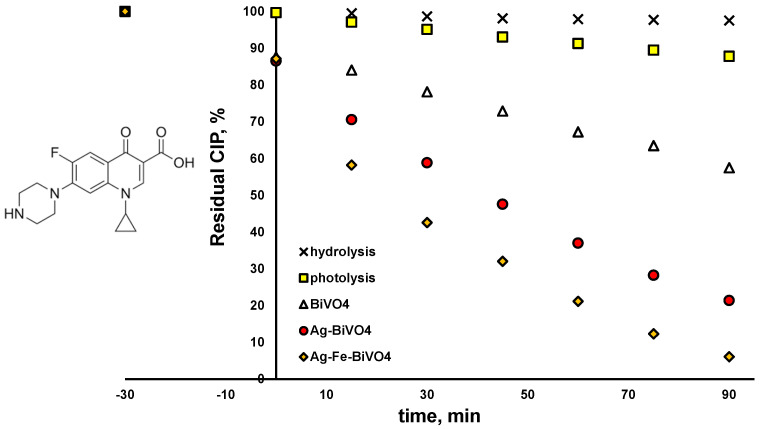
Degradation profiles of ciprofloxacin (CIP) using different photocatalytic materials: pristine BiVO_4_, Ag–BiVO_4_, and Ag–Fe–BiVO_4_, as well as hydrolysis and photolysis under studied conditions: solar light, natural pH (5.5), γ (catalyst dose) = 1 g/L (where applicable).

The activity of the studied BiVO_4_ materials (pristine and doped analogues (Ag–VO_4_ and Ag–Fe–BiVO_4_)) toward selected CECs, CIP, and PFOA was examined under solar irradiation. Besides photocatalytic degradation, both CECs were tested for their susceptibility to be removed either by photolysis (under solar irradiation) or due to hydrolysis. As can be seen from Figure 6, CIP is susceptible to photolysis; 12.2% was degraded within 90 min by exposure to induced solar irradiation. The plausible explanation can be found in the fact that CIP absorbs light in the UV-A region as well [49], which is part of the solar spectrum. The same effect may enable CIP hydrolysis as well, which can also be promoted by extreme pH or elevated temperature [50]. Very moderate hydrolysis occurred in our case (1.5% removal in 90 min). Besides direct removal by photolysis and hydrolysis, removal due to adsorption on studied materials was analyzed as well, performing experiments in the dark (no solar irradiation) over 120 min periods. The obtained results were not shown due to the fact that adsorption–desorption equilibrium was achieved within a 30 min dark period, which proceeded later to photocatalytic experiments. Hence, as can be seen from Figure 6, all three studied photocatalytic materials showed similar adsorption ability toward CIP; 12.6–13.4% of CIP was removed due to adsorption during dark initial periods regardless of the BiVO_4_ type applied. As can be also seen from Figure 6, all three BiVO_4_ materials studied were able to degrade CIP; however, with different levels of effectiveness. Hence, pristine BiVO_4_ yielded 42.5% of degraded CIP after 90 min of treatment under solar irradiation, while two surface-decorated analogues showed improved activity toward CIP removal; 78.6% and even 93.9% in the cases of Ag–BiVO_4_ and Ag–Fe–BiVO_4_ photocatalysts, respectively. As mentioned above, structural properties and morphology were preserved in comparison to pristine material, while only slight changes occurred in band gap sizes. However, the biggest difference was obtained in the ability to suppress recombination of photogenerated charges, enabling higher photoactivity of surface-decorated materials compared to the pristine analogue. Furthermore, modeling actions showed that HOMO-LUMO energy is lowered in the cases of surface-decorated materials, suggesting that they have higher photoactivity as well. Based on the band gap size and the position of E_FB,_ the material demonstrates the ability to maintain oxidative reactions leading to ^●^OH generation, which facilitates the degradation of CECs (i.e., CIP) that undergo preferably oxidative degradation.

**Figure 7:**
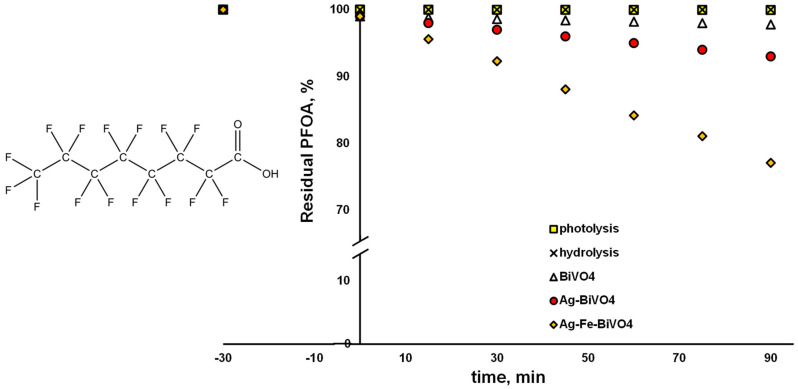
Degradation profiles of perfluorooctanoic acid (PFOA) using different photocatalytic materials: pristine BiVO_4_, Ag–BiVO_4_, and Ag–Fe–BiVO_4_, as well as hydrolysis and photolysis under studied conditions: solar light, natural pH (5.5), γ (catalyst dose) = 1 g/L (where applicable).

However, on the other hand, in cases where the same materials were applied for the degradation of PFOA and CEC undergoing preferably reductive degradation via O_2_^●−^, different results were obtained (Figure 7). Pristine and Ag-decorated materials showed very limited ability to degrade PFOA over a 90 min course, yielding 2.2% and 6.9% degradation, respectively. Somewhat better performance was observed in the case of Ag–Fe–BiVO_4_ (22.9% removal); however, the conversion results were much lower in comparison to those obtained for CIP. The reason may be that PFOA is a highly persistent chemical, which preferably undergoes a reductive pathway of degradation, yielding C–F cleavage, which can be accomplished by *e*^−^ or even by O_2_^●−^ [37,51]. However, *E*_FB_ of our surface-decorated BiVO_4_ materials is not suitable to generate O_2_^●−^, while degradation over photogenerated *e*^−^ cannot be expected due to the fact that PFOA adsorption onto BiVO_4_ materials is negligible (Figure 7); <1% was removed during the initial dark period. Thus, only bulk reactions can be expected. The degraded portion of PFOA in the cases of Ag–BiVO_4_ and especially Ag–Fe–BiVO_4_ can be assigned to cleavage of C–C bonds [52], which can be accomplished by generated ^●^OH. However, such a scenario would not contribute to the elimination of perfluorinated compounds (such as PFOA by-products), which is highly desired. Besides showing persistence to photogenerated radical species, PFOA showed resistivity to photolysis under solar irradiation and hydrolysis as well. The reason can be found in the fact that PFOA absorbs light within the low UV region (<220 nm), while it is highly resistant to hydrolysis, with a half-life > 92 years [53].

**Figure 8:**
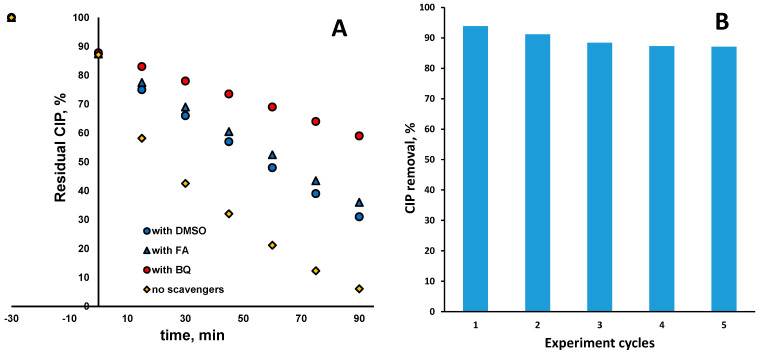
CIP removal in the presence of the common scavenging agents BQ, DMSO, and FA (**A**) and cyclic experiments to test stability of Ag–Fe–BiVO_4_ photocatalyst (solar light, natural pH (5.5), γ (catalyst dose) = 1 g/L (where applicable)) (**B**).

The involvement of the generated radical species HO• and O_2_^•−^, as well as photogenerated charge carriers (primarily *h*^+^) in bulk and direct degradation of CIP, respectively, was studied in common scavenging tests employing DMSO, BQ, and FA as scavenging agents, respectively. The results are shown in Figure 8A. As can be seen, the main degradation mechanism of CIP is reductive, since the major suppression in CIP degradation occurred in the case with BQ addition, even at 52.7%. It should be noted that involvement of *e*^−^ in direct reductive CIP degradation should be counted as well, perhaps largely due to the calculated position of E_FB_ and the fact that CIP is adsorbed during dark periods onto photocatalysts in amounts of approx. 12.5% (Figure 8A). However, the involvement of the oxidative mechanism, either directly over *h*^+^ or in bulk over HO•, cannot be neglected either because CIP degradation was also lowered noticeably in those cases by 29.8 and 25.1%, respectively. In order to test the stability of the Ag–Fe–BiVO_4_ photocatalyst, the cyclic experiments were performed; results are shown in Figure 8B. Hence, the same material was run over five cycles, exhibiting performance in slightly decreasing order of 93.1, 91.2, 88.4, 87.3, and 87.1% of CIP removal. The decrease in photocatalyst performance may be attributed to photocorrosion of silver structures at the catalyst surface, which may be stabilized after formation of Ag–O–Ag [54]. This presumably occurred within the last three cycles due to the fact that the values reached steady states.

## 3. Materials and Methods

### 3.1. Chemicals

Chemicals used for preparation of catalyst and its surface-decorated forms: bismuth nitrate pentahydrate (Bi(NO_3_)_3_ × 5H_2_O), ethylenediaminetetraacetic acid disodium salt dihydrate (EDTA-2Na), ammonium metavanadate (NH_4_VO_3_), nitric acid (HNO_3_), silver nitrate (AgNO_3_), and iron (III) chloride hexahydrate (FeCl_3_ × 6H_2_O) were purchased from VWR Chemicals (Vienna, Austria), T.T.T. (Sveta Nedelja, Croatia), Across Organics (Geel, Belgium), Lachner (Neratovice, Czech Republic), and Kemika (Samobor, Croatia), respectively. Representatives of CECs used in the study (CIP and PFOA) were both purchased from Across Organics (Geel, Belgium). Milli-Q water was used throughout the synthesis and degradation experiments. All reagents were utilized as received without further purification.

### 3.2. Experimental Procedure

#### 3.2.1. Preparation of BiVO_4_

In a typical experiment, an appropriate amount of Bi(NO_3_)_3_ × 5H_2_O (0.2 M) was dissolved in 1.5 M HNO_3_. To achieve this, 0.382 g of EDTA-2Na was added and the mixture was stirred continuously for 30 min to ensure complete dissolution (solution A). Meanwhile, solution B was prepared by dissolving NH_4_VO_3_ (0.2 M) in hot mill-Q water. Thereafter, solution A was added drop wise into solution B under vigorous stirring, this addition resulted in the formation of an intense orange–yellow colored suspension. The mixture was then allowed to stir in the dark for a period of one week. The resulting precipitate was collected by centrifugation and washed several times with water. The collected slurry was then dried at 80 °C in a hot-air laboratory oven. Once dried, the yellow solid was finely ground using a mortar and pestle. Finally, the solid powder was calcined at 450 °C for 2 h in a muffle furnace.

#### 3.2.2. Photomodification of BiVO_4_ by Ag and Fe

Photomodification of BiVO_4_ was achieved by incorporating silver (Ag) and iron (Fe) under light irradiation to synthesize Ag–BiVO_4_, and Ag–Fe–BiVO_4_ combinations. To prepare these materials, a weighed amount of as-synthesized pristine BiVO_4_ (10 g/L) was dispersed in a mixed solvent of water and ethanol (80:20). An optimum amount of AgNO_3_ and FeCl_3_ × 6H_2_O was added to the dispersion to achieve the desired weight percentages of Ag (1%) in Ag–BiVO_4_ and both Ag and Fe (6 and 18%, respectively) in Ag–Fe–BiVO_4_. The dispersion was then subjected under constant illumination using an unfiltered 450 W xenon arc lamp (XBO 450 W OFR, Osram, Munich, Germany) housed in an Oriel/Newport arc lamp system (Irvine, CA, USA) for 60 min. After the irradiation process, the resulting samples were collected via centrifugation and thoroughly washed and dried following the same protocol used for pristine BiVO_4_.

### 3.3. Photocatalytic Procedures

Photocatalytic degradation experiments were conducted in a glass reactor equipped with a circular water-cooling jacket, under the solar simulator featuring a collimator and an AM 1.5 G air mass filter. A 450 W Xenon lamp housed in research-grade arc lamp housing (Oriel/Newport Corporation, Irvine, CA, USA) served as the light source. The reactor was filled with 90 mL of either a PFOA (5 ppm, i.e., 12 μM) solution or a CIP (16.5 ppm, i.e., 50 μM) solution, each containing 90 mg of photocatalyst (γ = 1 g L^−1^). Prior to illumination, the suspension was stirred in the dark for 30 min to establish adsorption–desorption equilibrium. During the experiment, the suspensions were exposed to light, and 1 mL samples were withdrawn at regular intervals. These samples were filtered through 0.45 μm cellulose syringe filters (Chromafil, Xtra CA, Macherey-Nagel, Dueren Germany) and submitted to analysis by chromatographic methods (described below). The reaction mechanisms affecting CIP degradation by Ag–Fe–BiVO_4_ were studied by scavenging reactive oxygen species/sites generated during the early stages of photocatalysis. For this purpose, 1.0 mM of 1,4-benzoquinone (C_6_H_4_O_2_, BQ, 98%, Fluka, Buchs, Switzerland), 10 mM of formic acid (FA, VWR Chemicals, Lutterworth, UK), and 10 mM of dimethyl sulfoxide ((CH_3_)_2_SO, DMSO, 99.9%, Sigma Aldrich, Saint Louis, MO, USA) were used as superoxide anion radicals (O_2_^•−^), photogenerated holes (*h*^+^), and HO• scavengers, respectively. All experiments were conducted in triplicate and average values were reported; the reproducibility of experiments calculated based on LC–MS/MS and HPLC measurements was 97.9%.

### 3.4. Characterization

The thorough structural and morphological characterization of Ag–BiVO_4_ was conducted in our previous study [30], while here we inspected Ag–Fe–BiVO_4_ for crystal structure and phase compositions by a MiniFlex 600 X-ray diffractometer (Rigaku, Japan) with copper Kα radiation using a D/teX Ultra detector (Rigaku, Tokyo, Japan) in XRF suppression mode. The accelerating voltage and applied current were set at 40 kV and 10 mA, respectively. Furthermore, the same material was submitted for surface morphology and elemental composition inspection, performed by field emission scanning electron microscopy (FE-SEM, Zeiss, Jena, Germany) along with energy dispersive X-ray spectroscopy (EDS). Both Ag–BiVO_4_ and Ag–Fe–BiVO_4_ samples, along with pristine BiVO_4_ were submitted to thorough analysis of their optoelectronic properties, performing diffuse reflectance spectra (DRS) and photoluminescence (PL) spectra analyses, along with various electrochemical characterization tests. DRS analysis was conducted on immobilized samples on glass substrates that were analyzed using a 2600i UV-Vis spectrophotometer equipped with an ISR-2600Plus integrating sphere (Shimadzu, Kyoto, Japan). The recorded spectra were plotted as Tauc plots using Kubelka–Munk transformation [55]. PL analysis was conducted at room temperature using a Varian Cary Eclipse fluorescence spectrophotometer (Agilent, Santa Clara, CA, USA) with excitation wavelengths between 395 and 430 nm.

Electrochemical characterization was conducted to assess the electronic properties and current responses of the samples. For this purpose, working electrodes were fabricated by sequentially depositing three layers of the photocatalyst (Ag–Fe–BiVO_4_) onto a fluorine-doped tin oxide (FTO) glass substrate. The electrochemical measurements for CIP were performed in a standard three-electrode configuration, where the Ag–Fe–BiVO_4_-coated FTO glass served as the working electrode, a platinum (Pt) wire was employed as the counter electrode, and a saturated calomel electrode (SCE) was used as the reference electrode. In the case of PFOA, the measurements were performed using two-electrode systems, with an Ag–Fe–BiVO_4_-coated FTO plate acting as a working electrode, and platinum (Pt) as a counter electrode. All measurements were completed using an SP-150 potentiostat/galvanostat (Biologic, Seyssinet-Pariset, France) and an LED light source (COB, 50 W, 4400 lm, 6000 K) [24] in a solution of sodium sulphate (0.5 M, Na_2_SO_4_, Sigma-Aldrich, Taufkirchen, Germany) or Na_2_SO_4_ with the addition of CIP or PFOA. Open circuit potential (OCP) and linear sweep voltammetry (LSV) with a scan rate of 20 mV s^−1^ were performed under LED illumination and in the dark.

Concentrations of selected CECs, PFOA, and CIP were analyzed using liquid chromatography–tandem mass spectrometry (LC–MS/MS, LCMS-8045, Shimadzu, Kyoto, Japan) and high-performance liquid chromatography (HPLC, LC-20, Shimadzu, Kyoto, Japan), respectively. CIP concentrations were analyzed using an isocratic elution method. The mobile phase consisted of an 80:20 (*v*/*v*) mixture of aqueous 0.1% FA and methanol (J.T. Baker, Radnor, PA, USA). Separation was achieved on a reverse-phase Atlantis T3 C18 column (150 mm × 4.6 mm, 5 µm particle size; Waters, Milford, MA, USA). CIP detection was performed at its characteristic wavelength of 270 nm. Data acquisition and processing were carried out using LabSolutions 5.97 SP1 software (Shimadzu, Kyoto, Japan). To determine the concentration of PFOA in LC–MS/MS, the mobile phase comprised 70:30 (*v*/*v*) mixtures of 20 mM aqueous ammonium acetate (VWR Chemicals, Lutterworth, UK) and methanol (Fisher chemicals, Waltham, MA, USA) delivered at a flow rate of 0.4 mL min^−1^ under isocratic conditions for 10 min. The injection volume was 5 μL. Chromatographic separation was achieved on a shim-pack GIST C18 column (2.1 I.D. × 150 mm, Shimadzu, Kyoto, Japan) which was thermostated at 40 °C. The mass spectrometer operated in negative ion mode, recording the molecular ion of PFOA (M-H)^−^ at 413 in the first quadrupole (Q1). The second quadrupole monitored the characteristic ion fragments using multiple reaction monitoring (MRM) transitions: 413→369, 413→219, and 413→169 *m*/*z*. The collision energy was set at 20 eV. Nitrogen was used as the nebulizing gas (flow: 3 L/min) and drying gas (flow: 5 L/min), while argon served as the collision gas. Instrument conditions included an interface temperature of 190 °C, desolvation line temperature of 200 °C, and heating block temperature of 300 °C.

### 3.5. Modeling Procedure

Our theoretical modeling approach is based on the concept of a continuous random network. We view BiVO_4_ compounds as amorphous materials because the addition of certain elements, such as silver and iron, to the surface of the BiVO_4_ crystal disrupts its crystallinity. In such an approach, we looked at clusters composed of BiVO_4_ units. We used the program Gaussian 09/16 and within it the density functional theory (DFT) method with the B3LYP functional and the LANL2DZ basis set. For each cluster of n-units, we calculated its optimal geometry, which means that all vibrational frequencies of the cluster are strictly positive. The clusters doped with solely Ag or Fe were defined as having a net charge of 0, whereas the Ag and Fe co-doped cluster was defined to have a net charge of +1 for the sake of maintaining a closed electron shell configuration. The partial density of states (PDOS) was analyzed using Multiwfn 3.8 software [56,57] The binding energy per atom was determined using the following Equation (1):

(1) $$E_{\mathrm{bind}}=\frac{E\left({\left({\mathrm{BiVO}}_{4}\right)}_{n}\right)-n\times \left[E\left(\mathrm{Bi}\right)+E\left(V\right)+4\times E\left(O\right)\right]-E\times n\times \left(\mathrm{heteroatoms}\right)}{n\left(\mathrm{cluster}\right)}$$

where *E*_bind_ is the binding energy in eV, while *E*(Bi), *E*(V), and *E*(O) are the respective atomization energies, *E*(heteroatoms) corresponds to atomization energies of Fe or Ag, and *n* corresponds to the number of atoms.

## 4. Conclusions

In summary, we have successfully prepared the iso-type homojunction of pristine BiVO_4_ decorated by Ag and Fe. The materials were analyzed through various characterization techniques, such as XRD, SEM, UV-DRS, PL, and photoelectrochemical (PEC) tests. A theoretical HOMO-LUMO modeling approach was followed to match the experimental results. The modeling results revealed that doping with Ag and Fe lowered the LUMO, especially in Ag–BiVO_4_, where the LUMO electron state fell within the unmodified HOMO-LUMO gap for BiVO_4_. This narrowing of band gaps increases the reduction potential of photo-generated electrons, improving the photocatalytic capacity and providing valuable theoretical insights into the material’s enhanced performance. A slight narrowing of the band gap (by 0.1 eV) was observed in the modified Ag–Fe–BiVO_4_ compared to pristine BiVO_4_, as indicated by the DRS results as well. Lower PL intensity of doped analogues of BiVO_4_ (i.e., Ag–BiVO_4_ and Ag–Fe–BiVO_4_) demonstrated greater separation of photogenerated *e*^−^/*h*^+^, which facilitates the photoactivity of the doped materials. Further, PEC tests revealed high photoactivity of Ag–Fe–BiVO_4_, as a significant increase in the photocurrent was observed under the light. Moreover, a slight increase in current response with CIP indicates its electrochemical oxidation, while no such current difference was observed with or without PFOA, validating its stability in the oxidation process.

The photocatalytic activity of pristine BiVO_4_ and its doped analogues was tested using CIP and PFOA as model CECs. It was found that Ag–Fe–BiVO_4_ is the most efficient photocatalyst at degrading both CECs; CIP degradation reached 93.9%, while PFOA reached only 22.9%. The large differences in degradation rates can be attributed to the different preferred degradation pathways of CIP and PFOA. The band gap size and *E*_FB_ of the material indicates the capacity for oxidative generation of ●OH, which facilitates the degradation of CIP through the oxidative pathway. However, in the case of PFOA, which is even more persistent due to its strong C–F bonds, degradation preferably occurs through the reductive pathway involving O_2_^●−^ or via direct *e*^−^ transfer. Nevertheless, the position of *E*_FB_ is not favorable for efficient generation of O_2_^●−^, and electron-mediated degradation was negligible due to the poor adsorption of PFOA on the surface of BiVO_4_. Therefore, the observed degradation of PFOA can be attributed solely to the cleavage of C–C bonds facilitated by ●OH. However, in the case of CIP, the occurrence of the reductive degradation pathway was proven. The as-prepared co-doped photocatalyst showed moderate stability through repetitive cycles.

## Figures and Tables

**Table 1:** HOMO-LUMO gap energy calculated for studied BiVO_4_ materials.

Number of Units of BiVO_4_ (n)	(BiVO_4_)_n_ HOMO-LUMO Gap in eV	(BiVO_4_)_n_ + 2Ag HOMO-LUMO Gap in eV	(BiVO_4_)_n_ + 2Fe HOMO-LUMO Gap in eV	(BiVO_4_)_n_ + Ag + Fe HOMO-LUMO Gap in eV
1	3.81	1.89	2.67	1.88
2	3.90	1.62	2.78	2.02
3	2.21	2.23	2.79	2.44
4	2.86	1.52	1.43	
5	3.77	1.25		
6	3.79	1.37		
7	3.64	1.49		
8	2.61	1.93		
9	3.74	1.10		
10	3.38	2.54		
11	3.17	1.37		
12	3.29	2.76		
13	3.16	1.18		
**Average**	**3.33 (+/−0.5)**	**1.72 (+/−0.5) (13)**	**2.41 (+/−0.6)**	**2.11 (+/−0.3)**

## Data Availability

The datasets collected and analyzed in this work are available from the corresponding authors upon reasonable written request.
